# Machine learning models for classification tasks related to drug safety

**DOI:** 10.1007/s11030-021-10239-x

**Published:** 2021-06-10

**Authors:** Anita Rácz, Dávid Bajusz, Ramón Alain Miranda-Quintana, Károly Héberger

**Affiliations:** 1 Plasma Chemistry Research Group, Research Centre for Natural Sciences, Magyar tudósok krt. 2, Budapest, 1117 Hungary; 2 Medicinal Chemistry Research Group, Research Centre for Natural Sciences, Magyar tudósok krt. 2, Budapest, 1117 Hungary; 3 Department of Chemistry and Quantum Theory Project, University of Florida, Gainesville, FL 32603 USA

**Keywords:** ADMET, Toxicity, Big data, QSAR, In silico modeling, Machine learning

## Abstract

In this review, we outline the current trends in the field of machine learning-driven classification studies related to ADME (absorption, distribution, metabolism and excretion) and toxicity endpoints from the past six years (2015–2021). The study focuses only on classification models with large datasets (i.e. more than a thousand compounds). A comprehensive literature search and meta-analysis was carried out for nine different targets: hERG-mediated cardiotoxicity, blood–brain barrier penetration, permeability glycoprotein (P-gp) substrate/inhibitor, cytochrome P450 enzyme family, acute oral toxicity, mutagenicity, carcinogenicity, respiratory toxicity and irritation/corrosion. The comparison of the best classification models was targeted to reveal the differences between machine learning algorithms and modeling types, endpoint-specific performances, dataset sizes and the different validation protocols. Based on the evaluation of the data, we can say that tree-based algorithms are (still) dominating the field, with consensus modeling being an increasing trend in drug safety predictions. Although one can already find classification models with great performances to hERG-mediated cardiotoxicity and the isoenzymes of the cytochrome P450 enzyme family, these targets are still central to ADMET-related research efforts.

**Supplementary Information:**

The online version contains supplementary material available at 10.1007/s11030-021-10239-x.

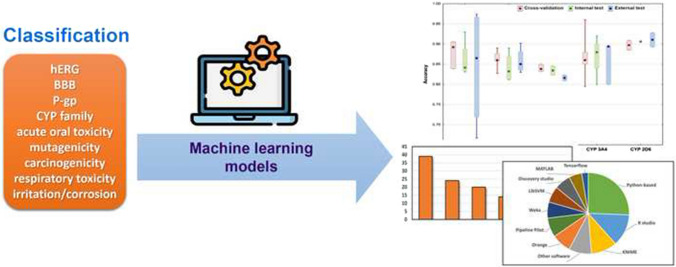

## Introduction

In the past decade, machine learning (ML) has undergone a definite revival in connection to the emergence of big data and the increase of compute capacities. Some maintain that big data have the potential to challenge the scientific method itself for new discoveries in science, through studying data correlations at a large scale [1]. Either way, present computer facilities allow us to analyze larger and larger data sets. As the computer power (speed and amount of data) increased, machine learning algorithms proliferated and artificial neural networks (ANNs) achieved a renaissance, their key role being further underlined by the appearance of deep learning methods to handle previously unprecedented amounts of data [2]. A consolidated description of big data is given by integrating definitions from practitioners and academics, and mainly deals with analytics related to unstructured data, which constitute 95% of big data [3].

In computer-aided drug design, the application of machine learning methods constitutes the new generation of QSAR modeling, although with the larger amount of training data (wider applicability domain) most authors aim for developing classification, rather than regression models. Recently, Maran et al. have reviewed a large amount of QSAR articles (1 533) on 79 individual endpoints of environmental and medicinal chemistry relevance, from all years up until 2015 [4]. From this plethora of QSAR studies 1235 contained multiple linear regression (MLR) modeling, 226 ANN, 77 support vector machines (SVM), 42 k-nearest neighbors (kNN), 39 decision trees (DT), 35 random forests (RF) and a few others. On one hand, it shows the unchallenged leading role of MLR in the previous decades of QSAR modeling; on the other hand, it reveals the significant effect of the machine learning revolution that is still going on.

Parallel to the unshaken popularity of linear modeling, an unprecedented proliferation of nonlinear machine learning algorithms took place. The present work, as well as other surveys show the most frequently used ML techniques: tree-based methods (e.g*.*, random forest, bagging, boosting and their variants), extensions of artificial neural networks (*e.g.*, deep leaning networks, DNN), support vector machines (SVM), *k*-nearest neighbors (*k*NN) and Naïve Bayes (NB). The latter two techniques are involved as standard, classical, well-known techniques mainly for benchmarking; on the other hand, SVM, tree-based algorithms and neural networks are trending now in all aspects of data science. ML algorithms are routinely used in (i) bioactivity [5], as well as property predictions of drug related compounds [6]; (ii) de novo drug design, *i.e*., generation of new chemical structures of practical interest [7]; (iii) virtual screening [8]; (iv) prediction of reaction pathways [9] and v) compound-protein interactions [10], etc. ML algorithms are mainly aimed at prediction, for which a great selection of descriptors and chemical representations, as well as many ML algorithms can be combined [11]. ML models are trained to recognize structural patterns that differentiate between active and inactive compounds. Understanding the reasons why models are so effective in prediction is a challenging task but of utmost importance to guide drug design [12].

As ML algorithms are easily overfitted, proper validation is of crucial importance. It is an eye-opening conclusion of the review of Maran et al*.* that reproducible studies (615) are in minority as compared the non-reproducible studies (882) [4]. Although there is no silver bullet that will always produce a reliable estimation of prediction error, a combination of cross-validation techniques achieves consolidated and superb performance in the prediction of unknowns. There are many known and accepted ways for the validation of ML models, such as i) randomization (permutation) tests [13]; ii) the many variants of cross-validation, such as row-wise, pattern-wise, Venetian blinds, contiguous blocks, etc.[14]*.*; iii) repeated double cross-validation [15] iv) internal and external test validation and others. A statistical comparison of cross-validation variants for classification was published recently [16].

ADMET (absorption, distribution, metabolism, excretion and toxicity) properties are crucial for drug design, as they can make or break (usually break) the career of drug candidates. Due to their central role, the present review will concentrate on collecting machine learning classification studies of ADMET-related targets in the last five years, providing a meta-analysis of nine important ADMET endpoints.

## Methods

In the past decades, artificial intelligence has escaped the world of science fiction and became a ubiquitous, albeit often hidden, part of our lives. While the self-definition of the field for intelligent agents (autonomous units capable of reacting to environmental changes for a specific goal) is very broad and includes such everyday devices as a simple thermostat, people usually associate artificial intelligence with more complex systems. A prime example for the latter is machine learning, which gradually became a dominating approach in many scientific areas including classification, especially in the case of large datasets. There are several trains of thought to machine learning models (see below), but probably the two most popular, “main” branches are tree-based and neural network-based algorithms. Deep learning methods are mostly neural networks of increased complexity, capable of handling unprecedented amounts of data; a few illustrative examples from the world ADMET endpoints highlight their potential for multitask modeling (predicting multiple endpoints simultaneously) [17, 18].

### Tree-based algorithms

Tree-based methods are very popular choices among machine learning techniques, not just in the field of ADME-related in silico modeling. The basic concept of tree-based algorithms is the use of decision trees for classification (and also regression) models. The trees are constructed in the following way: recursive binary splits are performed on the dataset based on the different features, parent and child nodes are created in this way, and the samples are separated into classes based on the majority class of the members in the terminal nodes (without child nodes) [19, 20].

There are new ensemble alternatives of the simple decision trees, such as random forests or gradient boosted trees. In the case of random forests (RT), one can use a voting-based combination of single decision trees for the classification of the objects with a better performance. Gradient boosting is an upgraded version, when the single decision trees are built sequentially with the boosting of the high performance ones and the minimization of the errors [21]. The optimized version of gradient boosted trees is the extreme gradient boosted tree (XGBoost) method, which can handle missing values and with a much smaller chance to overfitting. The tree-based algorithms are useful to handle complex nonlinear problems with imbalanced datasets, although in the case of noisy data they still tend to overfit. The hyperparameters (especially in XGBoost) should be tuned.

### Neural networks

Artificial neural networks (ANNs) and their specialized versions such as deep neural networks (DNN) or deep learning (DL) are one of the most common algorithms in the machine learning field, for ADMET-related and other prediction tasks [22, 23]. The basic concept of the algorithm is inspired by the structure of the human brain. Neural networks consist of input layers, hidden layer(s) and output layer(s). The hidden layers include a number of neurons. Every input variable in the input layer has different weights. A nonlinear activation function helps to transform the different linear combination of the input nodes into the output value. The weights are optimized in an iterative process to decrease the error of the prediction, for example with feed-forward or back propagation.

The major difference between traditional neural networks and deep learning is the amount of data and the complexity of the network. DL networks usually consists of several hidden layers, while classical neural networks are using usually just one (or two). In DL, the molecular descriptors are transformed into more abstract levels from layer to layer with the capability to manage complex functions. With such a complex network, overfitting is a possibility thus the network should be tuned [18]. The problem of overfitting is managed in deep neural networks with different improvements such as dropout [24]. Neural networks can be used for both regression and classification problems, and the algorithm can handle missing values and incomplete data. Probably, the biggest disadvantage of the method is the so-called “black-box” modeling; the user has little information on the exact role the provided inputs.

### Support vector machine

Support vector machines (SVM) are a classical nonlinear algorithm for classification and regression modeling as well. The basic idea is the nonlinear mapping of the features in a higher dimensional space. A hyperplane is constructed in this space, which can define the class boundaries. Finding the optimal hyperplane needs some training data, and the so-called support vectors [25]. For the optimal separation by the hyperplanes, one should use a kernel function such as a radial basis function, a sigmoidal or a polynomial function [26]. Support vector machines can be applied for binary and multiclass problems as well. SVM works well in high dimensional data and the kernel function is a great strength of the method, although the interpretation of the weights and impact of the variables is difficult.

### Naïve Bayes algorithms

Naïve Bayes algorithm is a supervised technique, which is based on the Bayesian theorem and the assumption of the uncorrelated (independent) features in the dataset. It also assumes that no hidden or latent variables influence the predictions (hence the name “naïve”) [27]. It is a simpler and faster algorithm compared to the other ML techniques; however, usually it has a cost in accuracy. Naïve Bayes algorithms are connected to Bayesian networks as well. Individual probability values for each class are calculated to every object separately. The naïve Bayes algorithm is very fast, even in the big data era compared to the other algorithms, but it performs better in the less complex and “ideal” cases.

### Nearest neighbor-based algorithms

The *k*-nearest neighbor algorithm is one of the simplest and most commonly used classification methods [28, 29]. Simple, because this method only needs the calculation of distances between the ligand pairs in the dataset. In the case of *k*-nearest neighbors, the algorithm considers the group of *k* nearest compounds (objects) and classifies the compounds/objects according to the majority votes in the class. Lots of variants are existing, such as N-nearest neighbors (N3), which is extended to all the *n*-1 compounds from *k*, or binned nearest neighbors (BNN) [30]. Nearest neighbor type algorithms can be used for binary and multiclass classification, and regression as well. These algorithms are easy to understand and intuitive, but they are sensitive to outliers and imbalanced datasets.

## ML models on the most prominent ADME targets

Several ADME and toxicity (ADMET) related endpoints have been selected for the comparative analysis of the last five years of research literature. Only classification models and categorical endpoints were selected, thus some important, but mostly regression-based models such as PAMPA or clearance are not covered by this review due to the different trends in these areas. Another focal point of this collection was to limit the considered studies to those with at least one thousand compounds. This way, we could provide a well-defined comparison among the trending algorithms and recent modeling habits.

### hERG-mediated cardiotoxicity

The human ether-à-go-go-related gene (hERG) encodes the α subunit of a voltage-gated potassium channel, which is one of the most important antitargets in drug discovery, as the inhibition of this ion channel results in fatal arrhythmia (sudden cardiac death) by prolonging the QT interval of cardiac action potential [31]. As such, significant research efforts are invested into screening compounds against hERG inhibition and developing predictive models to avoid compounds with hERG liabilities in the first place. Conventionally, hERG inhibition is evaluated in patch-clamp electrophysiological assays [32, 33], with thallium-flux assays being a relatively new alternative [34, 35]. The availability of large hERG inhibition datasets in PubChem Bioassay [36] and ChEMBL [37] allows for the development of reliable predictive models for hERG inhibition, with wide applicability domains.

Here, we have collected 15 works from the past five years that employ machine learning-based classification approaches to predict hERG inhibition [38–51]. All of these works apply training datasets of more than 1,000 molecules (and up to tens of thousands in some cases [47, 48]), and an overall majority presents two-class (active vs. inactive) classification (with the notable example of the 2015 study of Braga et al*.*, who have introduced a third class of “weak blockers”) [38]. Categorizing the molecules into the active and inactive classes is usually done by applying common activity thresholds such as 1 µM, 10 µM or their combination, a comprehensive methodological comparison was presented by Siramshetty et al. [44]*.* Indeed, most of these works use the PubChem and ChEMBL databases as the data source, with a few examples of literature sources or other databases (NCATS, GOSTAR, etc*.*). Independently of the choice of software and machine learning methods, classification performances are routinely great. The Comparative analysis section contains more details about the performance of the models.

### Blood–brain barrier penetration

The blood–brain barrier (BBB) is formed by brain capillary walls and glial cells to prevent harmful substances from entering the brain [52]. The penetration of this natural protective barrier of the central nervous system (CNS) by small molecules can be advantageous (in the case of CNS-directed drug candidates where BBB passage is a requirement of drug action) or disadvantageous. As such, measuring and predicting BBB penetration has been the focus of significant research efforts, particularly in CNS-related drug discovery [53]. As experimental data on BBB penetration is difficult to obtain, there are limited resources available for training machine learning models: there is relatively scarce data on BBB penetration in ChEMBL and PubChem Bioassay. Therefore, most studies rely on a limited number of core literature where experimental logBB (blood–brain distribution coefficient, log(*c*_brain_/*c*_blood_)!) values of altogether a few thousand compounds are collected [54, 55].

Here, we have collected seven machine learning classification studies from the past five years[56–62], with training sets of at least 1000 (and typically around 2000) compounds, employing popular machine learning methods such as random forests of support vector machines. All of these studies apply a two-class (penetrant or BBB + *vs*. non-penetrant or BBB–) classification scenario, usually with logBB thresholds of + 1, –1 or their combination. In addition to the most popular software choices and dedicated machine learning/deep learning platforms, 2D molecule images also appear as an interesting choice for compound descriptors in the work of Shi et al. [60]*.*

### Permeability glycoprotein (P-gp)

Permeability glycoprotein (P-gp) is a membrane protein that plays a pivotal role in the transport of a plethora of substrates through the cell membrane. This means that P-gp (which is expressed in blood–tissue and blood–brain barriers, among many other types of tissues like liver, colon, etc*.*) is of fundamental importance in pharmacokinetics, by regulating the efflux properties of a drug [63]. Coupled to ATP hydrolysis, P-gp can excrete several substrates out of the cell [64], this is why the over-expression of P-gp is a key factor in multidrug resistance [65]. Additionally, indiscriminate inhibition of P-gp in liver tissue will interfere with the excretion of xenobiotics [17], potentially leading to hepatotoxicity. All this explains why much effort has been devoted to the study of P-gp inhibitors and substrates.

P-gp substrates and inhibitors are usually tested in separate studies and naturally there are more studies with the focus on inhibitors [17, 66–72] instead of substrates [68, 73]. The use of consensus modeling for this endpoint seems to be a viable option, a good example is the work of Yang et al*.* [72]. In another specific study of Prachayasittikul and coworkers [70], the authors used SMILES-based descriptors to build a novel classification model using the CORAL software. The pseudo-regression model also shows great promise, with accuracy values over 80%, despite being relatively simple. Finally, among the most recent studies on P-gp inhibition we have the work of Esposito et al. [73], which uses molecular dynamics fingerprints as descriptors. Overall, all methods performed very well, even external validation accuracies were above 0.70. A detailed comparison will be presented in the Comparative analysis section.

### Cytochrome P450 enzyme family

The cytochrome P450 enzymes (CYP) have a crucial role in the metabolism of the xenobiotics. The CYP family of enzymes is also involved in drug safety and efficacy, because of the responsibility in drug-drug interactions (DDIs) [74]. In the human body, 57 different CYP isoforms can be found. Out of these, the most important six isoforms (CYP1A2, CYP2B6, CYP2C9, CYP2C19, CYP2D6 and CYP3A4) of the family metabolize more than 95% of the FDA-approved drugs [75].

In recent five years, several machine learning classification models have been developed for the mentioned targets [76–83]. There are several online data sources with experimental results (such as PubChem Bioassay) for the different isoenzymes separately and together as well. The classification models are strongly connected to the PubChem Bioassay database: these datasets were used for almost every model, with one exception [77]. In one specific case, namely the 2C9 isoform, the collected dataset has reached even 35 000 different molecules [74]. It should be emphasized, that the presence of the different CYP isoforms enables the development of multitarget classification models [80, 83]. The performances of the different models are discussed in detail later in the Comparative analysis section.

### Acute oral toxicity

Acute toxicity can be defined as oral, dermal or inhalation, but out of the three types, oral toxicity is the most well-known and thoroughly examined. It is an important endpoint from the early stage of drug discovery, since a compound that is hazardous for human health should be filtered out as early as possible [84]. Several machine learning models have been developed for the prediction of the median lethal dose (LD_50_) values of the compounds in continuous (regression) and categorical (classification) setups as well. Rodents are the most common animals to test the median lethal dose of a compound, thus the usual datasets for machine learning modeling contain this type of data.

In our study, we have summarized the relevant classification models [85–88]. Different guidelines help in the categorization of the compounds in the different toxicity classes, such as the four-class system of the U.S. Environmental Protection Agency (EPA) [89] or the five-class version of the United Nations Globally Harmonized System of Classification and Labelling (GHS) [90]. Although multiclass classification is more frequent, one can find two-class classifications too, where the datasets are separated into very toxic or non-toxic (positive and negative) classes [87]. For this endpoint, the datasets usually contain more than ten thousand compounds and consensus models are frequently used. More details about these models are discussed later in the Comparative analysis section.

### Carcinogenicity

Carcinogens are defined as chemical substances that can cause cancer and therefore, carcinogenicity is one of the most important toxicological endpoints, contributing even to the subsequent withdrawal of several approved drugs [91]. Carcinogenicity is usually tested in animal models [92], which, for ethical (and also economical) reasons, further underpins the importance of developing reliable predictive models to screen out potential carcinogenic liabilities early in the drug discovery process. As such, the prediction of carcinogenicity is the central topic of a vast literature, including early SAR and QSAR studies, and more recently, diverse machine learning approaches based on large training datasets [93–95]. It should be noted that structural alert-based systems can also achieve decent accuracies in carcinogenicity prediction [96], further supporting the use of molecular fingerprints in predictive models (as it was dominated in the corresponding literature data from the past five years). All the evaluated models for this target are based on the Carcinogenic Potency Database [97].

### Mutagenicity

Genetic toxicity testing is an early alternative of the carcinogenicity tests in the drug discovery processes. Bacterial tests are widespread methods in the pharma industry, and the *Salmonella*-reverse-mutation assay or Ames test is the in vitro gold standard for the task [98]. The Ames assay was developed by Bruce Ames and his colleagues almost fifty years ago [99], and still this is the most important assay for the determination of the mutagenic potential of compounds. Most of the online mutagenicity databases are based on this in vitro experiment.

In the past five years, several machine learning classification models have been developed for this endpoint [43, 100–103]. Most of them have applied six to seven thousand compounds for binary classification, mainly based on the Hansen Ames *Salmonella* mutagenicity benchmark data [104]. The performances were usually a bit lower compared to the other endpoints, especially in binary classification (see more details in the Comparative analysis section).

### Respiratory toxicity

Chemical respiratory toxicity can cause serious harm for the human body; moreover, the effects are not always obvious in the early stages [105]. Respiratory toxicity can lead to symptoms such as asthma, bronchitis, pneumonia, rhinitis, etc. Unfortunately, pulmonary drug toxicity is possibly an underdiagnosed cause of lung diseases. Therefore, it is also a major endpoint in ADMET studies. Naturally, in silico models can be useful alternatives to the usually applied animal experiments for the determination of respiratory toxicity.

We can find less publications compared to the other targets in the past few years [106–108], but performances are excellent for this endpoint. It is also worth to note that the size of the datasets is much smaller compared to other endpoints. Some commonly used and publicly available databases from the publications are ChemIDplus (TOXNET) (chem.nlm.nih.gov/chemidplus/), PNEUMOTOX (www.pneumotox.com) and ADrecs [109].

### Irritation/corrosion

Another important topic is the examination of the skin and eye irritation effects of the different chemicals. REACH requirements should be fulfilled before a compound is entering the market (European legislation, (Regulation EC No 1907/2006)) [110]. This regulation includes the endpoints of skin and eye irritation and serious damage (corrosion). Corrosive compounds can destroy the living tissues in the contact area (irreversible damage), while the irritative substances can cause inflammation (reversible damage) [111].

In this review, we have focused only on eye irritation. Eye irritation and corrosion experiments involve animal testing, preferably rabbits, but in silico approaches could potentially reduce the amount of animal testing in this case as well [110]. We have found three binary classification models from the past five years with more than one thousand compounds in the datasets [112, 113]. Gathering data for these endpoints is harder compared to other targets: usually several databases and literature data were merged into the final datasets for modeling.

## Comparative analysis

In this review, 89 different models were evaluated from the relevant literature as a representative set. It is worth mentioning that only those relevant ADME and toxicity targets were used, where the potential use of classification models is supported, *i.e.*, the target variable is categorical, such as inhibitor *vs*. non-inhibitor, toxic *vs*. non-toxic, etc. Our aim was to provide a comparison from the relevant publications of the last five years, when the authors used machine learning techniques in a combined or single mode for predicting different ADME-related endpoints in the big data era. The so-called “big data” formalism means different dataset sizes in science; thus, here we considered only those publications for the comparative study, where the datasets contained more than 1000 molecules. The gathering of the publications was closed on February 28, 2021. *The final database of the models is shown in the Supplementary material.*

**Fig. 1:**
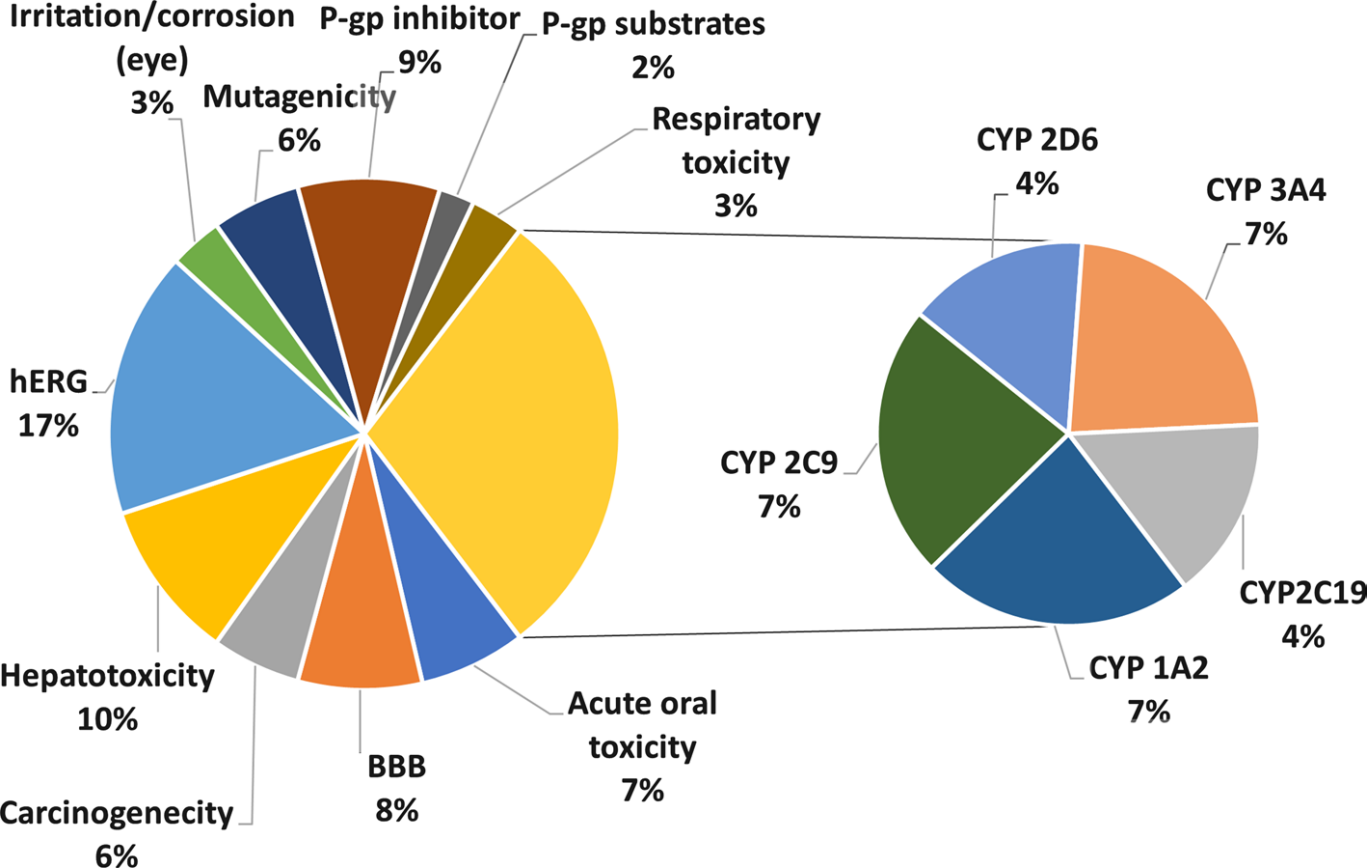
Distribution of the targets with percentages (BBB: blood–brain barrier)

Figure 1 shows the distribution among the different targets in the literature dataset. The CYP P450 isoforms (1A2, 2C9, 2C19, 2D6 and 3A4) were treated separately.

In the last five years in machine learning driven in silico classification modeling, the most frequent target was the drug metabolism related cytochrome P450 enzyme family. The distribution is closely uniform for the different isoforms, which can be attributed to the commonly used multi-targets in CYP P450 modeling. Another large proportion (17%) is connected to hERG (cardiotoxicity) modeling, since this target has a crucial role in drug safety as an antitarget and nowadays it is a routine procedure to test compounds for hERG-channel activity in the early stage of drug discovery.

Usually, more than one model is published in the papers, thus it is important to emphasize that only the best model for each target was evaluated from the publications in the following comparison.

The models were compared based on (i) the applied machine learning algorithm, (ii) the validation protocol, (iii) the used descriptor set, (iv) the modeling type (as consensus/single), (v) the performance of the models and (vi) the dataset size. Naturally, the authors did not always provide these parameters, thus missing values can occur in the dataset.

Consensus modeling means that the model was based on more than one machine learning algorithms and the authors applied various kinds of data fusion options for the development of the consensus model. It was interesting to see that 80% of the models were based on a single algorithm. As consensus modeling is a very common field of in silico machine learning, we have no doubt about the increase of this type of models in the near future, especially for more complex targets.

**Fig. 2:**
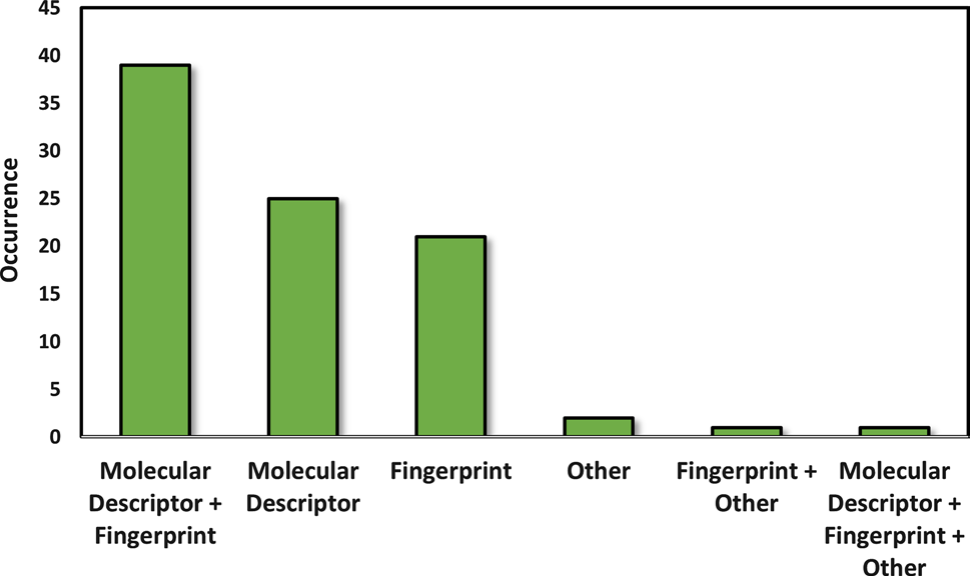
Occurrences of different descriptor types in the classification models

In QSAR/QSPR modeling, the use of different molecular descriptors, fingerprint variants and other X variables, such as docking score values or molecular dynamics simulation related variables has an important role. Several commercial software and publicly available tools offer the calculation of thousands of descriptors, and the selection of the appropriate ones can have a great effect in the final performance of the models. In Fig. 2, we have collected the used descriptor sets in the best models.

The most frequent combination was the application of classical 1D/2D/3D molecular descriptors with different fingerprints, which was followed by using only molecular descriptors and only fingerprints. Other descriptors, such as SMILES string related descriptors, molecular dynamics (MD) descriptors, 2D molecule images or docking score values are less frequently used, both alone and in combination with the other two favorite types.

**Fig. 3:**
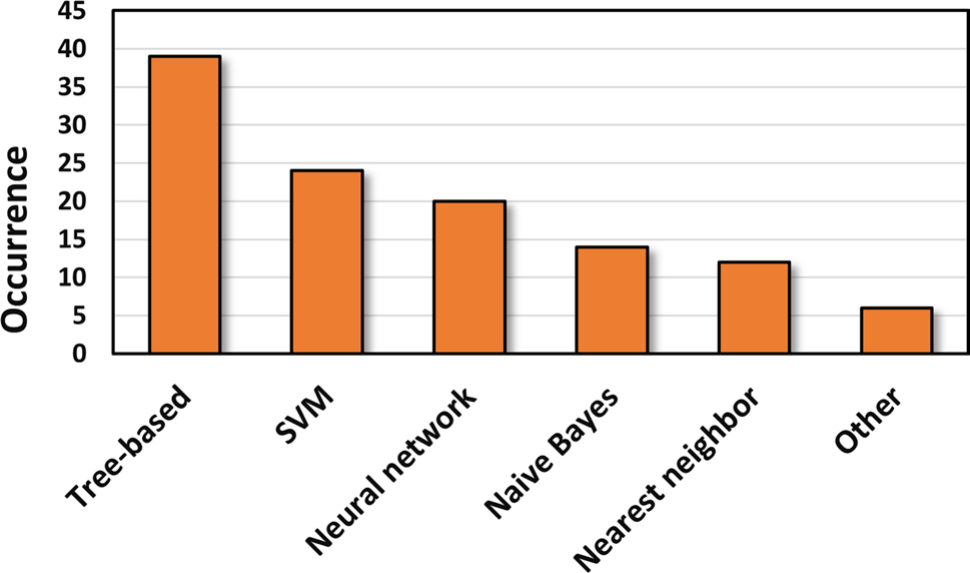
Occurrences of the different machine learning models in the collected dataset

Figure 3 shows the occurrences of the different machine learning algorithms. We have classified them into six different groups: tree-based algorithms such as random forests, XGBoost, etc.; neural networks, which includes every algorithm with different network systems; support vector machine-based algorithms; nearest neighbor-based algorithms, such as *k*NN, 3NN, etc.; Naïve Bayes algorithms; and the rest of them was classified as “Other”. It is important to mention that in the consensus models, all of the used algorithms were classified into the related groups, thus the sum of the occurrences is higher than 89. (If the authors used more than one algorithm from the same type in a consensus model, it was counted only once.)

Tree-based algorithms have clearly dominated in silico classification modeling in the ADME world in the past five years. SVM and neural network-based algorithms are also very common, and only a little amount of models contained algorithms other than the first five group, like logistic regression, LDA, self-organizing maps, SIMCA, etc. [72, 86, 114].

**Fig. 4:**
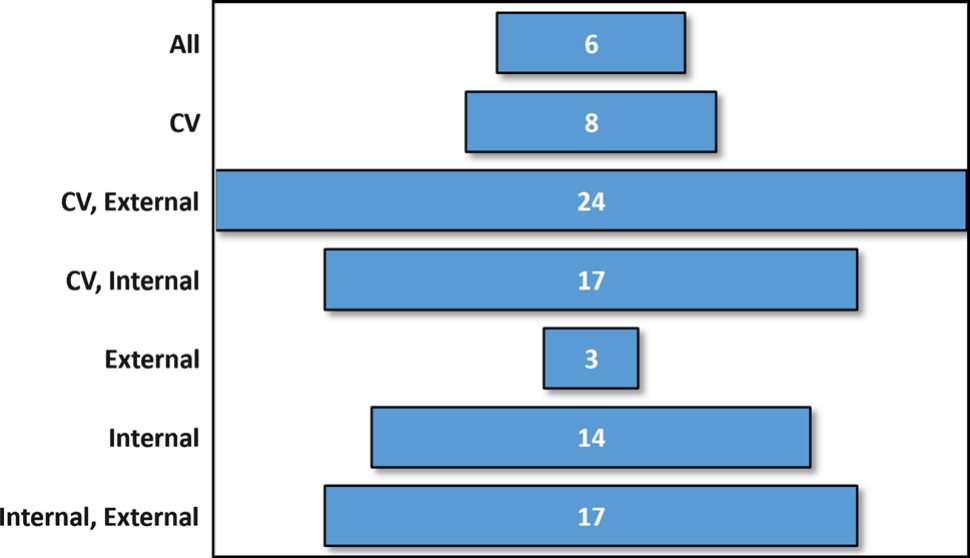
Occurrences of the different types of validations alone and in combination

The use of different validation practices for the verification of the models was a divisive factor among the selected publications. We have checked the application of cross-validation (*n*-fold), internal validation and external validation alone, and in combination. Internal validation meant that the originally used database was split into two parts (training and test), while external validation meant that the authors used another database for the external verification of the model. Moreover, the training-test set splits were also evaluated when internal validation was used. Figure 4 shows the application of the validation types in the publications.

It is clear that only a relatively small number of publications used all three type of validation. In most cases, cross-validation was used in combination with external test validation. However, it is surprising that in fourteen cases, only internal validation was used, which is at least a questionable practice. Three models were validated only externally, which is also interesting, because without internal or cross-validation, it does not reveal possible overfitting problems. Similar problems can be the use of only cross-validation, because in this case we do not know anything about model performance on “new” test samples.

**Fig. 5:**
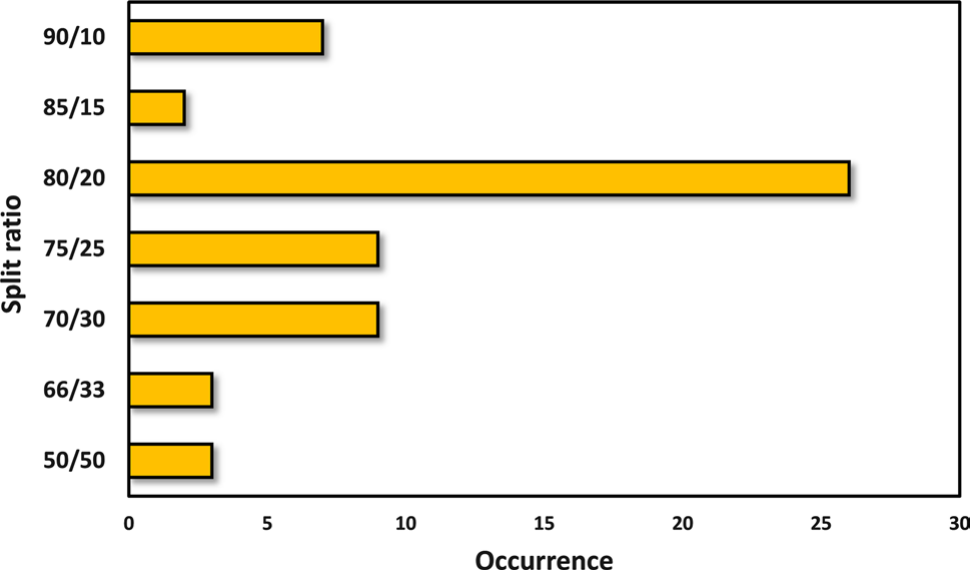
Occurrences of different split ratios in the train/test split of the datasets

Those models, where an internal validation set was used in any combination, were further analyzed based on the train–test splits (Fig. 5).

Most of the internal test validations used the 80/20 ratio for train/test splitting, which is in good agreement with our recent study about the optimal training-test split ratios [115]. Other common choices are the 75/25 and 70/30 ratios, and relatively few datasets were split in half. It is common sense that the more data we use for training, the better performance we have–up to certain limits.

**Fig. 6:**
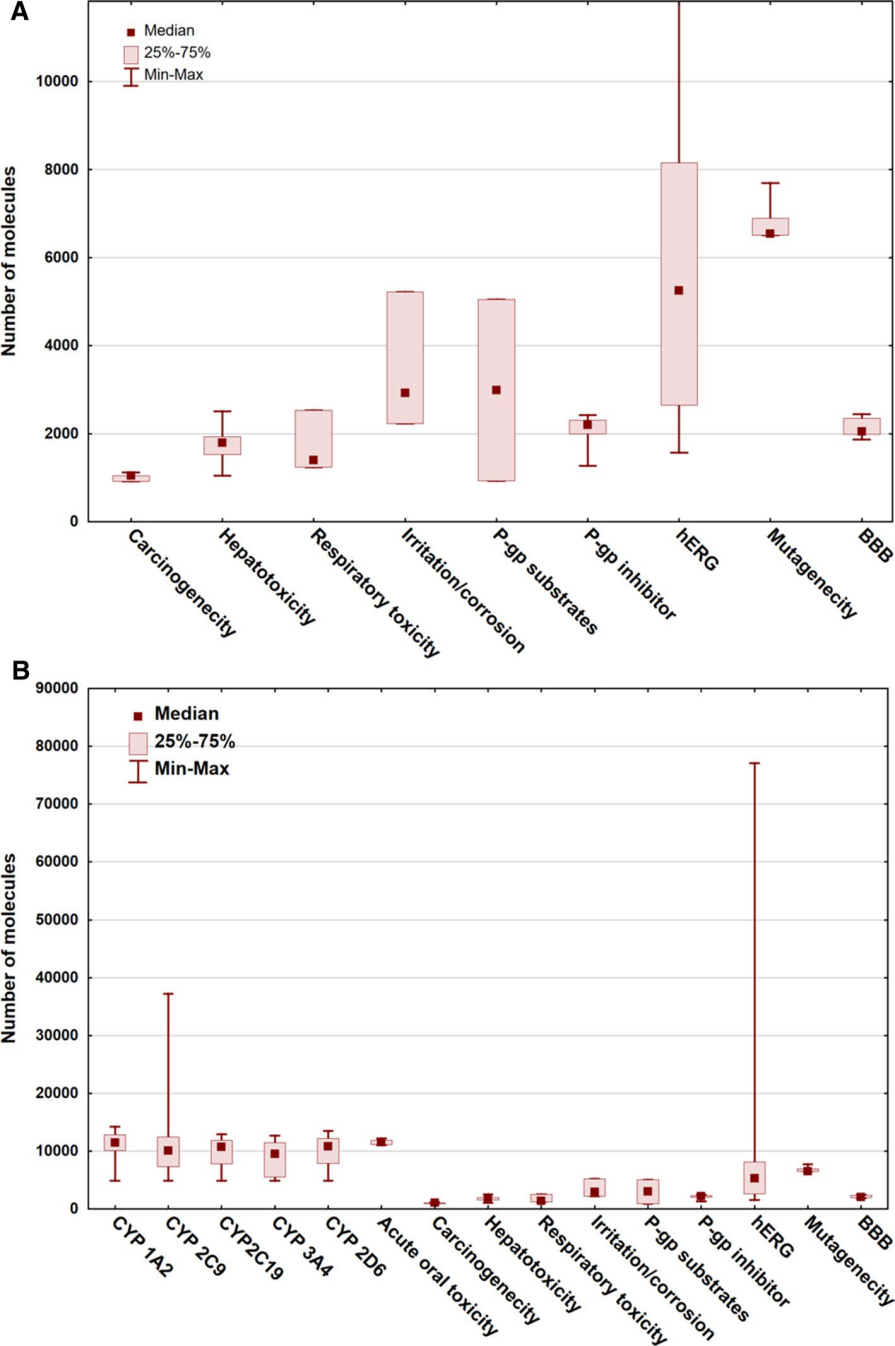
Dataset sizes for each examined target. Figure 6 **A** is the zoomed version of Fig. 6**B**, which is visually better for the targets with smaller dataset sizes. The number of molecules are plotted with the use of median, minimum and maximum values

The dataset size was also an interesting factor in the comparison. Even though we had a lower limit of 1000 compounds, we wanted to check the amount of the available data for the examined targets in the past few years. (We did one exception in the case of carcinogenicity, where a publication with 916 compounds was kept in the database, because there was a rather limited number of publications from the last five years in that case.) External test sets were added to the sizes of the datasets. Figure 6 shows the dataset sizes in a Box and Whisker plot with median, maximum and minimum values for each target.

The largest databases belong to the hERG target, while the smallest amount of data is connected to carcinogenicity. We can safely say that the different CYP isoforms, acute oral toxicity, hERG and mutagenicity are the most covered targets. On the other hand, it is an interesting observation that most models operate in the range between 2000 and 10,000 compounds.

**Fig. 7:**
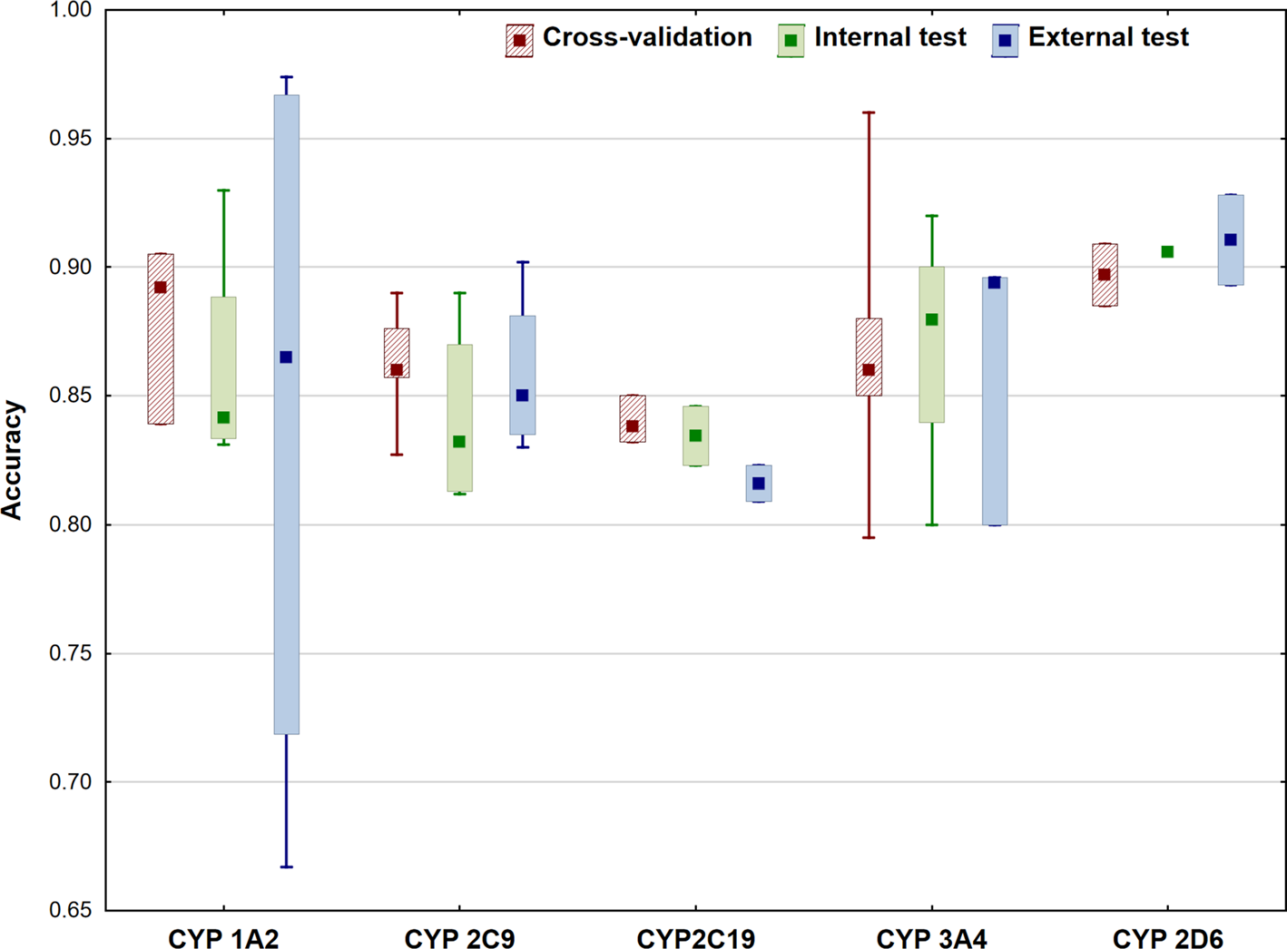
Comparison of the accuracies for the different classification models for CYP P450 isoforms. Median, minimum and maximum values are plotted for each target

**Fig. 8:**
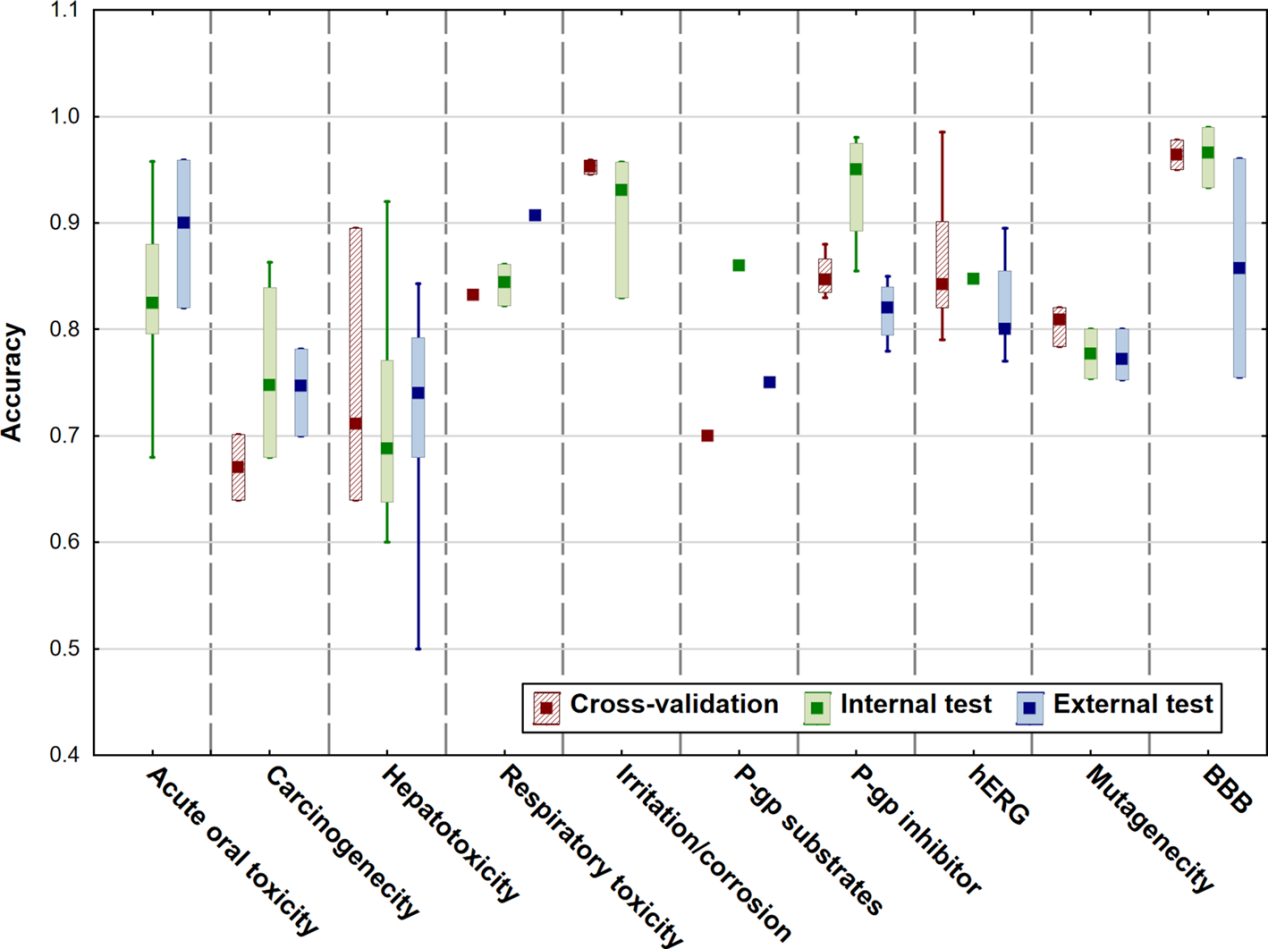
Comparison of the accuracies for the different ADME related targets. Median, minimum and maximum values are plotted

In the last section, we have evaluated the performance of the models for each target. Accuracy values were used for the analysis, which were not always given: in a few cases, only AUC, sensitivity or specificity values were determined, these were excluded from the comparisons. While accuracies were selected as the most common performance parameter, we know that model performance is not necessarily captured by only one metric. Figures 7 and 8 show the comparison of the accuracy values for cross-validation, internal validation and external validation separately. CYP P450 isoforms are plotted in Fig. 7, while Fig. 8 shows the rest of the targets.

For CYP targets, it is interesting to see that the accuracy of external validation has a larger range compared to internal and cross-validation, especially for the 1A2 isoform. However, dataset sizes were very close to each other in these cases, so it seems that this has no significant effect on model performance. Overall, accuracies are usually above 0.8, which is appropriate for this type of models. In Fig. 8, the variability is much larger. While the accuracies for blood brain barrier (BBB), irritation/corrosion (eye), P-gp inhibitor and hERG targets are very good, sometimes above 0.9, carcinogenicity and hepatotoxicity still need some improvement in the performance of the models. Moreover, hepatotoxicity has the largest range of accuracies for the models compared to the others.

Average accuracies were compared with ANOVA analysis to show the effect of the different machine learning algorithms (only single models with one machine learning algorithm were included). Moreover, average absolute differences of the accuracies were calculated between CV and internal validation, CV and external validation and between external and internal validation (where it was possible). ANOVA analysis was also carried out on these values, which could present the difference in the robustness between the algorithms. Nearest neighbors algorithm was excluded from the comparison, because it was used only in consensus modeling.

**Fig. 9:**
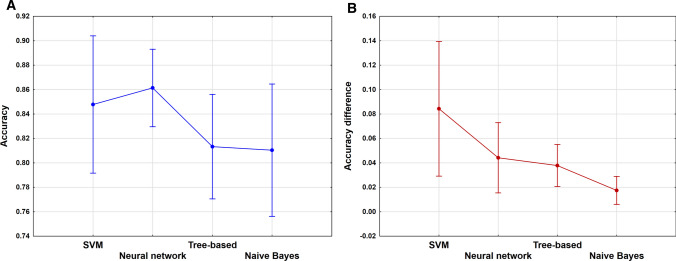
**a** **b** ANOVA analysis based on the **a** average accuracies and **b** average absolute differences of the accuracies. Machine learning algorithms are plotted in the X axis. The mean values and the 95% confidence intervals are shown in the figures.

Figure 9 shows the results of ANOVA. The machine learning algorithms have no significant effect on the models, but we have to note, that the variances are a bit bigger compared to the target related accuracies, due to the use of average values. On the other hand, in the case of the average absolute differences of the accuracies (b) a significant effect could be detected between the algorithms. We can observe that SVM and Neural networks have somewhat better average accuracies, but their robustness is worse compared to the Tree-based and Naïve Bayes algorithms.

**Fig. 10:**
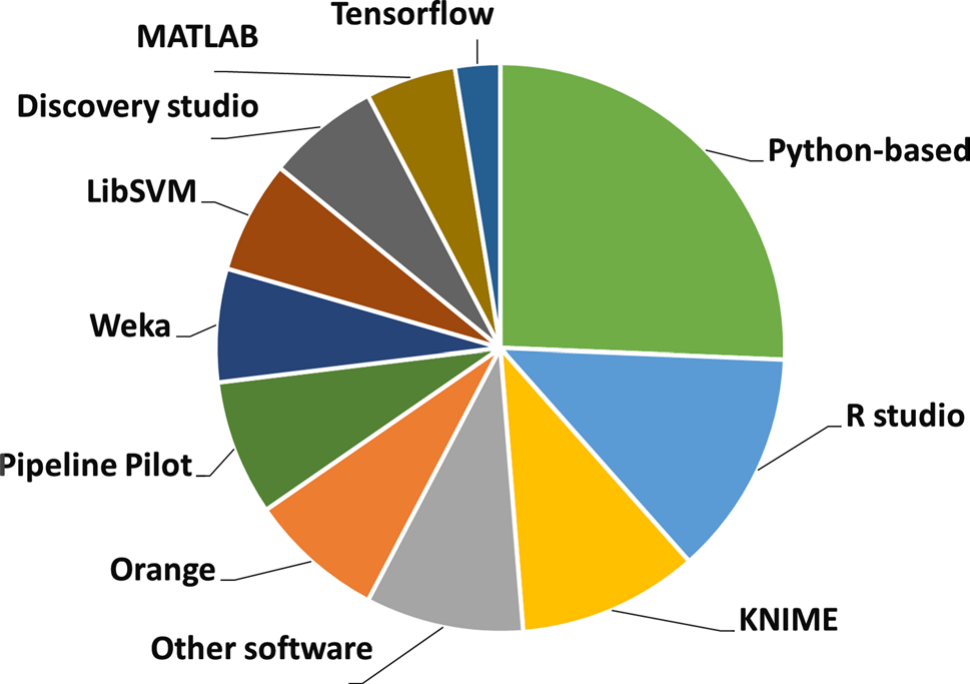
Comparison of the applied software packages

## Resources

In the past decades, the role of the different programming languages and open-source platforms in QSAR/QSPR modeling rapidly increased. Thus, it is not surprising that in the last five years, the most popular algorithms are connected to Python or R-based packages (see Fig. 10). One can find several machine learning packages for both platforms, however KNIME as a visual JAVA-based platform is also in this competition, because of the useful machine learning-related packages developed especially for classification problems. Several Python-based algorithms have KNIME implementations as well. One good example for this is Weka, which is also a well-known machine learning toolkit [116].

We have compared the software/platform usages in our dataset, where the authors shared this information. LibSVM (https://www.csie.ntu.edu.tw/~cjlin/libsvm/), Weka (https://www.cs.waikato.ac.nz/ml/weka/) and Tensorflow (www.tensorflow.org) software have several implementation options, thus we have decided to present these separately. Sometimes the authors have used more than one platform: these results are added separately to each segment. Almost half of the machine learning model developments are connected to either Python, R studio or KNIME. It is also worth to note, that Orange became a well-known open-source platform in the last couple of years [117]. Naturally, commercial software such as MATLAB or Discovery Studio are covering a smaller portion. Other software includes all the standalone developments (open-source or commercial) such as ADMET predictor (Simulations Plus, Inc., www.simulations-plus.com), PgpRules [68], CORAL [70] or Clementine (SPSS Inc., http://www.spss.com). The latter ones had usually single occurrences in the dataset.

We cannot overlook several useful web-accessible tools for ADMET predictions, such as ADMETlab (http://admet.scbdd.com) [118] or CypReact (https://bitbucket.org/Leon_Ti/cypreact) [119], which are also based on several machine learning models, although this is not the main focus of this review.

## Concluding remarks

The prediction of ADMET-related properties plays an important role in drug design as safety endpoints, and it seems that it will stay in this position for a long time. Several of these drug safety targets are connected to harmful or deadly animal experiments, raising ethical concerns, moreover, the cost of most of these measurements is rather high. Thus, the use of in silico QSAR/QSPR models to overcome the problematic aspects of drug safety related experiments is highly supported.

The use of machine learning (artificial intelligence) algorithms is a great opportunity in the QSAR/QSPR world for the reliable prediction of bioactivities on new and complex targets. Naturally, the increasing amount of publicly accessible data is also helping to provide more reliable and extensively applied models. In this review, we have focused on those models, which were based on bigger datasets (above one thousand molecules), to provide a comprehensive evaluation of the recent years’ ADMET-related models in the larger dataset segment. The findings showed the popularity of tree-based algorithms for classification problems. In the aspect of validation, many models still rely on only cross-validation or only internal validation, which signifies a room for improvement in validation practices. The in silico predictions of ADMET parameters have been, and will remain a central question of computational drug discovery and with the increasing databases, fast and efficient open-source platforms for modeling and the development of novel algorithms, we believe that dedicated machine learning models have proven to be indispensable tools.

## Supplementary Information

Below is the link to the electronic supplementary material.

Supplementary file1 (XLSX 22 KB)
